# Circulating TNF Receptors 1 and 2 Are Associated with the Severity of Renal Interstitial Fibrosis in IgA Nephropathy

**DOI:** 10.1371/journal.pone.0122212

**Published:** 2015-04-10

**Authors:** Yuji Sonoda, Tomohito Gohda, Yusuke Suzuki, Keisuke Omote, Masanori Ishizaka, Joe Matsuoka, Yasuhiko Tomino

**Affiliations:** 1 Division of Nephrology, Department of Internal Medicine, Juntendo University Faculty of Medicine, Tokyo, Japan; 2 Clinical Research Center, Juntendo University Faculty of Medicine, Tokyo, Japan; University of Tokushima, JAPAN

## Abstract

The current study aimed to examine whether the levels of TNF receptors 1 and 2 (TNFR1 and TNFR2) in serum and urine were associated with other markers of kidney injury and renal histological findings, including TNFR expression, in IgA nephropathy (IgAN). The levels of the parameters of interest were measured by immunoassay in 106 biopsy-proven IgAN patients using samples obtained immediately before renal biopsy and in 34 healthy subjects. Renal histological findings were evaluated using immunohistochemistry. The levels of serum TNFRs were higher in IgAN patients than in healthy subjects. The levels of both TNFRs in serum or urine were strongly correlated with each other (*r* > 0.9). Serum TNFR levels were positively correlated with the urinary protein to creatinine ratio (UPCR) and four markers of tubular damage of interest (N-acetyl-β-D-glucosaminidase [NAG], β2 microglobulin [β2m], liver-type fatty acid-binding protein [L-FABP], and kidney injury molecule-1 [KIM-1]) and negatively correlated with estimated glomerular filtration rate (eGFR). Patients in the highest tertile of serum TNFR levels showed more severe renal interstitial fibrosis than did those in the lowest or second tertiles. The tubulointerstitial TNFR2-, but not TNFR1-, positive area was significantly correlated with the serum levels of TNFRs and eGFR. Stepwise multiple regression analysis revealed that elevated serum TNFR1 or TNFR2 levels were a significant determinant of renal interstitial fibrosis after adjusting for eGFR, UPCR, and other markers of tubular damage. In conclusion, elevated serum TNFR levels were significantly associated with the severity of renal interstitial fibrosis in IgAN patients. However, the source of TNFRs in serum and urine remains unclear.

## Introduction

IgA nephropathy (IgAN) is the most common form of glomerulonephritis worldwide, and it presents with various histological and clinical phenotypes [1–3]. IgAN is characterized by the mesangial deposition of pathogenic polymeric IgA1, proliferation of mesangial cells, increased synthesis of extracellular matrix, and infiltration of macrophages, monocytes, and T cells. There is a strong correlation between the severity of renal interstitial damage and subsequent renal function decline in IgAN and diabetic nephropathy (DN) [4–6]. Chan *et al*. [7] have clearly demonstrated that TNFα released from mesangial cells after IgA deposition activates renal tubular cells and leads to subsequent inflammatory changes in the renal interstitium.

A number of studies have reported that the levels of circulating TNF pathway-related molecules, such as TNFα and TNF receptors (TNFRs), are significantly higher in chronic kidney disease (CKD) patients, and that these levels are closely correlated with changes in the estimated glomerular filtration rate (GFR) [8,9]. These biomarkers are also associated with lower GFR and higher albuminuria even in a community-based setting [10,11]. Moreover, results from the Joslin Kidney Study demonstrated that increased levels of circulating TNFR1 and TNFR2 emerged as very strong predictors of the progression of DN to CKD stage 3 or end-stage renal disease [12,13]. However, little is known regarding the association of circulating TNFRs or the urinary excretion of TNFRs with clinical and histological findings in IgAN patients. Therefore, we measured the levels of TNFRs in IgAN patients using serum and urine samples obtained immediately before renal biopsy and also examined renal histological findings, including TNFR expression.

## Materials and Methods

### Study patients and sample collection

The current study was conducted on 106 Japanese patients (53 men and 53 women, with a mean age of 35 ± 12 years) with newly diagnosed, biopsy-proven primary IgAN between 2005 and 2013. The control group consisted of 34 age-matched healthy subjects. Kidney tissue specimens were obtained using echo-guided percutaneous renal biopsy. Normal human kidney tissue samples were obtained from six nephrectomy specimens that were removed from renal tumors. Renal biopsy was usually performed in relatively less advanced cases, particularly when chronic glomerulonephritis was suspected, as shown in Table 1. We selected patients who had ≥nine glomeruli (median 21) for the valid diagnosis of IgAN. IgAN was diagnosed according to the mesangial deposition of polymeric IgA1, other immunoglobulins, and complements using immunofluorescence microscopy and electron-dense deposits in mesangial areas using electron microscopy. None of the enrolled patients had been treated with immunosuppressive drugs. Patients with secondary IgAN such as lupus nephritis, any concomitant disease, or symptoms of acute and chronic inflammation other than glomerulonephritis were excluded from the study. Serum and urine samples were collected from all patients immediately before renal biopsy and were stored at −80°C until use. The study protocol and informed written consent procedures were approved by the Institutional Review Board of Juntendo University Faculty of Medicine, Tokyo, Japan. Written informed consent was obtained from all participants, and the study adhered to the Declaration of Helsinki.

**Table 1:** Clinical characteristics and levels of inflammatory markers in IgAN patients and healthy subjects.

	IgAN patients (n = 106)	Healthy subjects (n = 34)	P
**Demographic Characteristics:**			
Male	53 (50.0%)	19 (55.9%)	0.55
Age (yr)	35±12	37±6	0.51
BMI (kg/m^2^)	21.7±2.9	21.6±2.2	0.81
SBP (mmHg)	113±13	118±15	0.19
DBP (mmHg)	66±9	75±13	0.003
ACEI or ARB Rx	18 (17.0%)	NA	
Uric acid (mg/dl)	5.9±1.5	NA	
Serum IgA (mg/dl)	311±107	NA	
Serum C3 (mg/dl)	97±17	NA	
IgA/C3 ratio	3.2±1.1	NA	
eGFR (ml/min/1.73m^2^)	79 (60, 100)	105 (93, 116)	<0.0001
eGFR categories:			
>90	35 (33.0%)	30 (88.2%)	
60–90	45 (42.5%)	4 (11.8%)	
30–60	21 (19.8%)	0 (0%)	
15–30	5 (4.7%)	0 (0%)	
UPCR (mg/g∙Cr)	402 (162, 972)	21 (16, 33)	<0.0001
Hematuria categories: (/HPF)			
1–5	3 (2.8%)	NA	
6–10	9 (8.5%)	NA	
11–20	25 (23.6%)	NA	
21–30	12 (11.3%)	NA	
≥31	57 (53.8%)	NA	
**Inflammatory markers:**			
Serum TNFR1 (pg/ml)	1412 (1264, 1807)	941 (834, 1017)	<0.0001
Serum TNFR2 (pg/ml)	2963 (2483, 3758)	2025 (1813, 2322)	<0.0001
Urinary TNFR1 (ng/g Cr)	1067 (727, 1609)	1469 (938, 2026)	0.06
Urinary TNFR2 (ng/g Cr)	2504 (1680, 3578)	2662 (1733, 3715)	0.58

Data are mean ± SD, median (quartiles), or %.

BMI, body mass index; SBP, systolic blood pressure; DBP, diastolic blood pressure; ACEI, angiotensin converting enzyme inhibitor; ARB, angiotensin receptor blocker; Rx, treatment; GFR, glomerular filtration ratio; UPCR, the ratio of urinary protein to creatinine; HPF, high power field; TNFR, TNF receptor; NA, not applicable

### Assessment of exposure variables

The parameters of interest were measured in the clinical laboratory at Juntendo University Hospital. The levels of serum creatinine (Cr) were measured using standard enzymatic methods [14]. eGFR was calculated using the modified Modification of Diet in Renal Disease (MDRD) equation proposed by the Japanese Society of Nephrology: eGFR (mL/min/1.73 m^2^) = 194 × [age (years)]^−0.287^ × [serum Cr (mg/dL)]^−1.094^ × 0.739 (for females). Urinary protein excretion was measured using a pyrogallol red-based reagent kit (Protein Assay Rapid Kit; Wako Pure Chemical Industries, Ltd., Osaka, Japan). The levels of urinary Cr were measured using an automated machine (Hitachi 7170S Chemistry Analyzer; Hitachi Chemical Co., Ltd., Tokyo, Japan) and a commercial kit (CRE-S; Denka Seiken Co., Ltd., Tokyo, Japan). Urinary protein excretion was expressed as mg/g∙Cr. Patient demographics and the other clinical parameters, including age, gender, body mass index, systolic and diastolic blood pressure (SBP, DBP, respectively), and the degree of hematuria, were recorded before renal biopsy. Medication history before renal biopsy, including the use of renin-angiotensin system blockers such as angiotensin-converting enzyme inhibitors and angiotensin receptor blockers, was also recorded.

### Measuring TNFRs, NAG, β2m, L-FABP, and KIM-1

The levels of soluble TNFR1 and TNFR2 in serum and urine were measured using an enzyme-linked immunosorbent assay (Cat # DRT100 and 200, R&D Systems, Minneapolis, MN, USA), as described previously [15]. The urinary levels of liver-type fatty acid-binding protein (L-FABP, Human L-FABP Assay Kit; CIMIC Co., Ltd., Tokyo, Japan) were measured using the enzyme-linked immunosorbent assay as reported [16,17]. The urinary levels of kidney injury molecule-1 (KIM-1; Cat # HKTX3MAG-38K, MILLIPLEX^@^ MAP Human Kidney Toxicity Magnetic Bead Panel 3–Toxicity Multiplex Assay; Merck Millipore, Austin, TX, USA) were measured using a multiplex assay run on the Luminex platform. This multiplex assay is a particle-enhanced, sandwich type, liquid-phase immunoassay with a laser-based detection system based on flow cytometry. All measurements were performed according to the manufacturer’s protocols.

Two internal serum controls were included in each assay to estimate the interassay coefficient of variation (CV). The interassay coefficient of variation for TNFRs and L-FABP was 0.7–10.0% (serum TNFR1, 0.7%; urinary TNFR1, 2.3%; serum TNFR2, 4.3%; urinary TNFR2, 10%; L-FABP, 3.3%), but it was higher for KIM-1 (22.0%).

The urinary levels of N-acetyl-β-D-glucosaminidase (NAG; Nittobo Medical Co., Ltd., Tokyo, Japan) and β2 microglobulin (β2m; Denka Seiken Co., Ltd.) were measured in the clinical laboratory at Juntendo University Hospital. Briefly, the enzymatic activity of NAG was measured using a colorimetric assay with 6-methyl-2-pyridyl-*N*-acetyl-1-thio-6-D-glucosaminide as the substrate, and β2m was measured using a latex aggregation assay kit. All urinary measurements were normalized individually to urinary Cr levels.

### Evaluation of renal histological findings

A nephrologist who was blinded to the patients’ clinical data evaluated the histological findings of each slide. The pathological characteristics of IgAN patients were evaluated, such as the proportion of interstitial fibrosis, tubular atrophy, and global and/or segmental glomerulosclerosis and proliferation of mesangial cells. Interstitial fibrosis and tubular atrophy were quantified using the computer-assisted imaging of tissue sections that had been stained with Masson’s Trichrome and Periodic acid-Schiff, respectively [18,19]. Image acquisition and analysis were performed using KS-400 version 4.0 (Carl Zeiss Vision, Munich, Germany). The entire cortical region of each renal biopsy was analyzed in a stepwise manner as a series of consecutive fields without overlapping. In each field, a region of interest was traced on the cortex excluding the glomeruli and large- or medium-sized blood vessels. Masson’s Trichrome-stained blue areas were recognized as regions of interstitial fibrosis. The total area of the cortical region of interest was then calculated, and the areas of interstitial fibrosis and tubular atrophy were quantified and expressed as a percentage of the cortex. Glomerulosclerosis (global and segmental) was calculated as the percentage of the total number of the glomeruli in a biopsy specimen. Mesangial hypercellularity was scored by counting the number of mesangial cells in the most cellular mesangial area of each glomerulus according to the Oxford classification [20,21]. Mesangial areas immediately adjacent to the vascular stalk were excluded. Then, the average score (mesangial cells/mesangial area) was calculated. Patients were classified into three groups according to the tertile of histological severity.

### Immunohistochemistry for TNFR1 and TNFR2 in the kidney

Immunohistochemistry was performed as described previously [22] using serial 3-μm sections of renal biopsy specimens. These tissues were dewaxed in xylene and then rehydrated in a graded series of ethanol. Endogenous peroxidase activity was inhibited by incubation in methanol containing 3% H_2_O_2_ for 10 min. The sections were then incubated with primary antibodies at 4°C overnight. Primary antibodies used were monoclonal mouse anti-TNFR1 (MAB225, R&D Systems) and monoclonal mouse anti-TNFR2 (MAB226, R&D Systems). The other stained sections were blocked using blocking solution (2% fetal bovine serum and 10% normal goat serum in PBS). The sections were then incubated with polyclonal HRP-labeled goat anti-mouse IgG anti serum (Dako, Carpinteria, CA, USA). Secondary antibodies were visualized by light microscopy using diaminobenzidine. For IgAN, we evaluated the entire renal cortex, including all glomeruli (median, 21; minimum, 12) and the tubulointerstitium. The entire cortical region of interest was calculated, and then, the TNFR-positive area was quantified using the KS-400 version 4.0 image analysis system and expressed as a percentage of the cortex. For control, we also evaluated all glomeruli (median, 24; minimum, 20) and the tubulointerstitium in 10 fields of the renal cortex using a microscope at 200× magnification.

### Statistical analysis

Continuous variables with a normal distribution are expressed as means ± SDs. Variables with a skewed distribution are presented as medians (25–75% interquartile range). Categorical variables are described as frequencies or percentages, and comparisons between groups were performed using Mantel–Haenszel χ^2^ tests for dichotomized variables. The Mann–Whitney U-test and Kruskal–Wallis test were used to assess differences between groups. Spearman’s regression analysis was used to analyze the correlation between two variables. Stepwise multivariate linear regression analysis was used to evaluate the independence of factors that showed significant correlation in the univariate model. A two-sided *P* value <0.05 was considered to be statistically significant. Statistical analyses were performed using SPSS software (version 19; SPSS Inc., Chicago, IL, USA).

## Results

### Clinical characteristics and levels of kidney injury markers in the study population

As shown in Table 1, the distribution of gender, age, and SBP did not differ between IgAN patients and healthy subjects. Although DBP was significantly higher in healthy subjects, the values were within the normal range. eGFR and the urinary protein to creatinine ratio (UPCR) were lower and higher, respectively, in IgAN patients, as expected. The levels of serum TNFRs were significantly higher in IgAN patients than in healthy subjects.

IgAN patients were divided into tertiles according to serum TNFR2 levels (Table 2). Age, UA, UPCR, and the prescription of renin-angiotensin system blockers were significantly different among tertiles; SBP was borderline insignificant. The levels of eGFR decreased significantly with an increase in serum TNFR2 levels. All markers of inflammation (serum and urinary TNFRs) and tubular damage (NAG, β2m, L-FABP, and KIM-1) increased with an increase in serum TNFR2 levels; similar results were observed among serum TNFR1 tertiles (data not shown).

**Table 2:** Clinical characteristics and levels of inflammatory and tubular damage markers according to tertile of serum TNFR2 levels in IgAN patients.

	Serum TNFR2			
	T1	T2	T3	
	(n = 35)	(n = 36)	(n = 35)	P
Demographic Characteristics:				
**Male**	17 (48.6%)	19 (52.8%)	17 (48.6%)	0.92
**Age (yr)**	34±10	30±10	42±12	0.001
**BMI (kg/m** ^**2**^ **)**	21.3±2.0	21.4±2.8	22.4±3.7	0.67
**SBP (mmHg)**	111±14	111±12	117±13	0.1
**DBP (mmHg)**	66±10	66±10	65±8	0.98
**ACEI or ARB Rx**	3 (8.6%)	4 (11.1%)	11 (31.4%)	0.02
**Uric acid (mg/dl)**	5.6±1.5	5.4±1.5	6.5±1.3	0.01
**Serum IgA (mg/dl)**	313±100	297±94	323±125	0.75
**Serum C3 (mg/dl)**	97±17	98±15	97±19	0.85
**IgA/C3 ratio**	3.3±1.1	3.1±0.9	3.4±1.2	0.63
**eGFR (ml/min/1.73m** ^**2**^ **)**	86 (70, 102)	86 (73, 106)	57 (37, 79)	<0.0001
**UPCR (mg/g∙Cr)**	263 (113, 708)	305 (135, 522)	963 (306, 1108)	0.001
Inflammatory markers:				
**Serum TNFR1 (pg/ml)**	1182 (1068, 1319)	1401 (1296, 1589)	2062 (1789, 2674)	<0.0001
**Serum TNFR2 (pg/ml)**	2372 (2222, 2483)	2963 (2757, 3164)	4198 (3744, 4783)	By design
**Urinary TNFR1 (ng/g∙Cr)**	866 (592, 1280)	1007 (575, 1302)	1390 (990, 2353)	0.001
**Urinary TNFR2 (ng/g∙Cr)**	1996 (1466, 2899)	2158 (1528, 3302)	3496 (2354, 4423)	0.002
Tubular damage markers:				
**NAG (IU/g∙Cr)**	4.5 (3.3, 6.2)	5.6 (3.5, 8.3)	8.8 (6.5, 11.6)	<0.0001
**β2m (μg/g∙Cr)**	69 (47, 99)	70 (43, 103)	121 (52, 327)	0.013
**L-FABP (μg/g∙Cr)**	2.7 (1.3, 4.2)	2.5 (1.4, 4.2)	4.7 (3.2, 7.7)	0.001
**KIM-1 (μg/g∙Cr)**	1.2 (0.6, 2.1)	1.2 (5.2, 2.5)	2.0 (1.3, 3.2)	0.009

Data are mean ± SD, median (quartiles), or %. Abbreviations used in this table are the same as in Table 1. NAG, N-acetyl-β-D-glucosaminidase; β2m, β2-microglobulin; L-FABP, liver-type fatty acid binding protein; KIM-1, kidney injury molecule-1.

### Association between markers of tubular damage or inflammation and impaired renal function

Markers of tubular damage or inflammation were studied by examining their correlations with each other, and with two renal function measures, eGFR and UPCR (Table 3). Significant negative correlations between all markers and eGFR, except KIM-1 and eGFR, were observed. Notably, only the correlation coefficient for serum TNFRs and eGFR exceeded 0.50. Interestingly, the correlation coefficients between the two TNFRs in serum or urine were >0.90, although correlations between serum TNFRs and urinary TNFRs were weak (*r* = 0.32–0.40). In addition, urinary TNFRs were strongly correlated with all markers of tubular damage, except NAG compared with serum TNFRs. There was a strong correlation (*r* = 0.71–0.73) between urinary TNFRs and L-FABP. However, the two renal function measures were more strongly correlated with serum TNFRs than with urinary TNFRs.

**Table 3:** Spearman correlation coefficients between markers of tubular damage or inflammation and impaired renal function in IgAN patients.

	UPCR	sTNFR1	sTNFR2	uTNFR1	uTNFR2	NAG	β2m	L-FABP	KIM-1
eGFR	-0.32^†^	-0.52^††^	-0.51^††^	-0.30**	-0.22*	-0.36^††^	-0.30**	-0.36^††^	-0.18
UPCR	1.00	0.42^††^	0.35^††^	0.17	0.17	0.55^††^	0.35^††^	0.48^††^	0.32^†^
sTNFR1		1.00	0.91^††^	0.40^††^	0.34^††^	0.49^††^	0.26**	0.40^††^	0.26**
sTNFR2			1.00	0.35^††^	0.32^†^	0.50^††^	0.28**	0.33^†^	0.26**
uTNFR1				1.00	0.94^††^	0.39^††^	0.44^††^	0.71^††^	0.47^††^
uTNFR2					1.00	0.38^††^	0.40^††^	0.73^††^	0.56^††^
NAG						1.00	0.36^††^	0.55^††^	0.53^††^
β2m							1.00	0.49^††^	0.30**
L-FABP								1.00	0.61^††^
KIM-1									1.00

*P<0.05

**P<0.01

†P<0.001

††P<0.0001

Abbreviations used in this table are the same as in Table 1 and 2. sTNFR, serum TNF receptor; uTNFR, urinary TNF receptor.

### Histological findings according to serum TNFR levels

**Fig 1:**
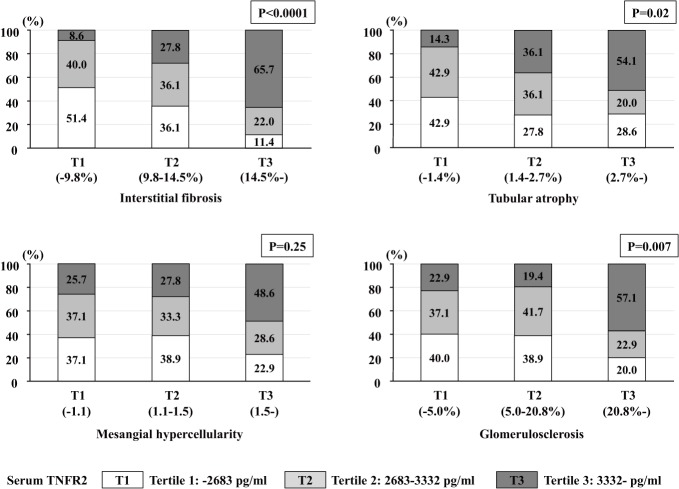
Histological findings according to serum TNFR2 levels. Patients were grouped according to distribution tertiles for each histological finding and serum TNFR2 levels. The severity of interstitial fibrosis, tubular atrophy, and glomerulosclerosis was significantly associated with serum TNFR2 levels.

To determine whether renal histology was associated with serum TNFR2 levels, patients were grouped according to the distribution tertiles of each histological finding. There was a significant relationship between serum TNFR2 levels and the percentage of interstitial fibrosis, tubular atrophy, or glomerulosclerosis in IgAN patients, whereas mesangial hypercellularity was not associated with serum TNFR2 levels (Fig 1). Interstitial fibrosis, tubular atrophy, and glomerulosclerosis worsened as serum TNFR2 levels increased. We also tried to examine renal histological findings by the MEST Oxford classification. However, we only analyzed segmental sclerosis and endocapillary hypercellularity, because mesangial hypercellularity (M1 > 0.5) was present in all patients, except two, and moderate to severe tubular atrophy/interstitial fibrosis (T2 > 50%, *n* = 0; T1: 26–50%, *n* = 3) was present in few patients. There was no association between serum TNFR2 and two histological findings (segmental sclerosis and endocapillary hypercellularity) [S0, 3021 (2511, 3639); S1, 2841 (2384, 4016); *P* = 0.74], [E0, 2887 (2417, 3720); E1, 3268 (2746, 3819); *P* = 0.23, respectively]. A similar pattern was observed with serum TNFR1 levels (data not shown).

### Association between interstitial fibrosis and serum TNFR levels

Table 4 shows the results of univariate and stepwise multiple regression analyses of the variables that were associated with renal interstitial fibrosis in IgAN patients. Univariate linear regression analysis showed that the percentage of interstitial fibrosis was significantly correlated with age (*r* = 0.42), SBP (*r* = 0.45), DBP (*r* = 0.31), UA (*r* = 0.45), eGFR (*r* = −0.60), UPCR (*r* = 0.54), and markers of inflammation (serum TNFR1, *r* = 0.58; serum TNFR2, *r* = 0.56; urinary TNFR1 *r* = 0.21) and tubular damage (NAG, *r* = 0.48; β2m, *r* = 0.25; L-FABP, *r* = 0.36; KIM-1, *r* = 0.19) (Model 1). Based on univariate regression analyses, stepwise multivariate regression analysis was then performed to examine the predictive potential of variables using interstitial fibrosis as the dependent variable. However, only serum TNFR1 or serum TNFR2 was included in the multivariate model because of the high colinearity between serum TNFR1 and serum TNFR2. Stepwise multivariate regression analysis revealed that serum TNFRs, eGFR, UPCR, and DBP could explain the percentage of interstitial fibrosis (Models 2 and 3). DBP and eGFR were common factors that could explain the severity of glomerulosclerosis, tubular atrophy, and interstitial fibrosis. In addition, UPCR and NAG were determinants for explaining the severity of glomerulosclerosis and tubular atrophy, respectively (data not shown).

**Table 4:** Simple and stepwise multiple regression analysis of variables that were associated with renal interstitial fibrosis in IgAN patients.

	Model 1	Model 2	Model 3
	Correlation	Standardized	
	coefficients	coefficients	
	r	β	β
Age	0.42^††^		
SBP	0.45^††^		
DBP	0.31^†^	0.15*	0.18*
Uric acid	0.45^††^		
eGFR	-0.60^††^	-0.28^††^	-0.30^††^
UPCR	0.54^††^	0.25^†^	0.26^†^
Serum TNFR1	0.58^††^	0.35^††^	
Serum TNFR2	0.56^††^		0.32^††^
Urinary TNFR1	0.21*		
Urinaru TNFR2	0.15		
NAG	0.48^††^		
β2m	0.25**		
L-FABP	0.36^††^		
KIM-1	0.19*		
		R = 0.75	R = 0.74
		Adjusted R^2^ = 0.54	Adjusted R^2^ = 0.53

*P<0.05

**P<0.01

^†^P<0.001

^††^P<0.0001

Abbreviations used in this table are the same as in Table 1 and 2.

### Immunohistochemical staining for TNFR1 and TNFR2 in the kidneys

Immunohistochemistry for TNFR1 and TNFR2 was performed with and without nuclear staining using serial renal biopsy specimens, because the TNFR-stained area seemed to be the nucleus. As shown in S1 Fig, performing immunohistochemistry without nuclear staining resulted in an unstained region that was expected to be the nucleus [TNFR1, B; TNFR2, E (arrows)]. The TNFR-stained area was also expected to be the nucleus [TNFR1, A; TNFR2, D (arrows)] and might represent staining in the supranucleus cytoplasm.

**Fig 2:**
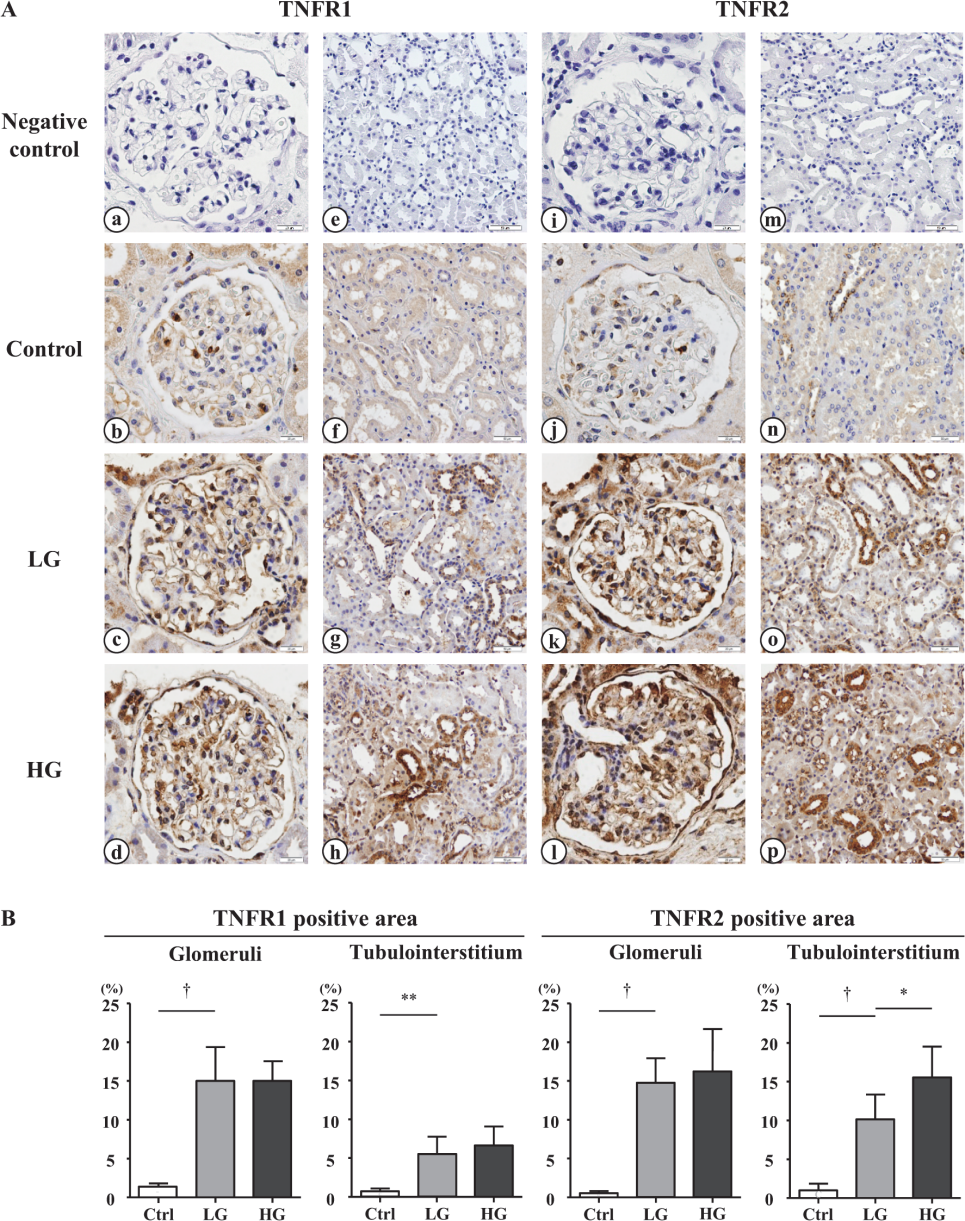
Representative immunohistochemical staining for TNFR1 and TNFR2 in the kidneys. (A) Images were captured at 200× (a, b, c, d, i, j, k, l) and 100× (e, f, g, h, m, n, o, p) magnification. Images show the negative controls in the glomeruli (a, i) and tubulointerstitium (e, m) and TNFR1 and TNFR2 immunostaining in the glomeruli (b, j) and tubulointerstitium (f, n) of the normal kidneys, respectively. TNFR1 and TNFR2 immunostaining is shown in the glomeruli (c, k) and tubulointerstitium (g, o) of the kidneys from selected IgAN patients who had levels of serum TNFR2 that ranked in the lowest 10 [low group (LG)]. TNFR1 and TNFR2 immunostaining in the glomeruli (d, l) and tubulointerstitium (h, p) of the kidneys from selected IgAN patients who had levels of serum TNFR2 that ranked in the highest 10 [high group (HG)], respectively, are also shown. (B) Percentage of the TNFR1 and TNFR2-positive area in the kidneys were evaluated. Glomerular and tubulointerstitial TNFR expression was elevated significantly in IgAN patients compared with those in control (Ctrl) subjects. The tubulointerstitial TNFR2-positive area was significantly larger in HG than in LG. However, there was no significant difference in the tubulointerstitial TNFR1 and glomerular TNFR areas between LG and HG. * *P* < 0.01, **P < 0.001, ^†^ *P* < 0.0001.

Ten patients whose biopsy specimens were available and who had levels of serum TNFR2 that ranked in the lowest 10 [low group (LG)] and highest 10 [high group (HG)] tertiles were selected for each group to assess whether serum TNFRs levels were associated with renal TNFR expression (S1 Table). Immunohistochemical staining for TNFRs was then performed, as shown in Fig 2A. In the normal kidney, TNFR1 was detected at weak levels in the glomeruli but was hardly detected in the tubulointerstitium. The TNFR1-positive area was increased in the glomeruli and tubulointerstitium in specimens from IgAN patients, but the percentage of each positive area was not significantly different between LG and HG (Fig 2B). In contrast, TNFR2 was hardly detected in the glomeruli in the normal kidney. There was faint staining for TNFR2 in the tubulointerstitium in the normal kidney, similar to TNFR1. The TNFR2-positive area was also increased in the glomeruli and tubulointerstitium in specimens from IgAN patients. The tubulointerstitial TNFR2-positive area was significantly higher in HG than in LG. As shown in S2 Table, serum TNFRs levels and eGFR were associated with tubulointerstitial TNFR2 expression but not tubulointerstitial TNFR1 expression.

## Discussion

Significant negative correlations between serum or urinary TNFR levels and eGFR and positive correlations between TNFR levels and UPCR were observed at the time of renal biopsy. In addition, elevated serum TNFR levels were associated with renal interstitial fibrosis in IgAN patients. This association remained significant even after adjusting for relevant clinical covariates such as age, UA, eGFR, and UPCR.

Previous studies demonstrated an association between TNFRs and various renal diseases [12,13,15,23–32], particularly DN [12,13,15,29–32]. However, little is known regarding the relationship between renal histological findings and the levels of TNFRs in serum and urine. To the best of our knowledge, this is the first study to demonstrate that high levels of serum TNFRs, but not urinary TNFRs, at the time of renal biopsy to reflect the severity of renal interstitial fibrosis in IgAN patients. Recently, Idasiak-Piechocka *et al*. [26] demonstrated that the levels of urinary TNFR1 were increased significantly in patients with various types of chronic glomerulonephritis, including ~35% of IgAN patients, compared with healthy subjects. Although we did not find such differences in the levels of urinary TNFRs between IgAN patients and healthy controls, serum TNFRs levels were significantly higher in IgAN patients than in healthy controls in the current study. This difference might result from the differences in renal function and proteinuria in the study populations. Specifically, Idasiak-Piechocka *et al*. analyzed patients with the renal disease that was more advanced than that in patients in the current study. In their study, the mean estimated Cr clearance using the Cockcroft–Gault formula was 75 ± 39 mL/min/1.73 m^2^, and the mean urinary protein excretion was 1.6 ± 1.5 g/day, even though 60% of patients had proteinuria in the non-nephrotic range. Although they also reported that both serum and urinary TNFRs were negatively associated with eGFR in IgAN patients, the correlation between urinary TNFRs and eGFR was weaker than that between serum TNFRs and eGFR in the current study. Taken together, this suggests that serum TNFRs might represent renal dysfunction more efficiently and sensitively than urinary TNFRs because serum TNFRs, but not urinary TNFRs, levels were already elevated in early-stage IgAN patients.

Fernández-Real *et al*. [29] reported that elevated serum TNFR1, but not TNFR2, levels were associated with renal interstitial fibrosis in patients with type 2 diabetes and the early stage of diabetic nephropathy; however, this relationship was no longer significant after controlling for age, body mass index, or SBP. In contrast, the present study demonstrated that the levels of both serum TNFRs were significantly associated with renal interstitial fibrosis in IgAN patients after the adjustment of multiple covariates. It is possible that these inconsistent results could be explained by differences in the underlying disease.

Each TNFR has distinct roles in inflammation and apoptosis/necroptosis [33,34]. However, the current study revealed that the strong correlation between serum TNFR1 and TNFR2 occurred even in patients with IgAN other than diabetic nephropathy (Spearman’s correlation coefficient *r* = 0.92) [15]. A similar observation was made with urinary TNFRs (Spearman’s correlation coefficient *r* = 0.94). An important consideration is the source of serum and urinary TNFRs. We first assumed that most TNFRs originated from injured kidneys. Immunohistochemical staining revealed that tubulointerstitial TNFR2 expression was associated with not only the severity of renal interstitial fibrosis but also serum TNFRs levels. However, tubulointerstitial TNFR1 and glomerular TNFR expression was not associated with either the severity of renal interstitial fibrosis or serum TNFRs levels. Because of the strong correlation between serum TNFR1 and TNFR2 levels (*r* = 0.92), tubulointerstitial TNFR2 expression was unlikely to affect serum TNFR2 levels, even though the increased tubulointerstitial TNFR2 expression affected interstitial fibrosis to a certain extent. Therefore, serum and urinary TNFRs were likely to be secreted from somewhere other than the kidneys. Interestingly, the fractional excretion of TNFR (FeTNFR) was increased with decreasing eGFR in the current study (data not shown).

Although glomerular dysfunction is a major factor for the development and progression of IgAN, interstitial damage might also play an important role in the pathophysiology and progression of IgAN [4–6]. In the current study, all markers of tubular damage (NAG, β2m, L-FABP, and KIM-1) were significantly associated with TNFR levels in serum and urine. In particular, there was a strong correlation between L-FABP and TNFR levels in serum and urine. We demonstrated previously that recombinant TNF-α upregulated L-FABP expression significantly in mouse proximal tubular cells that had been transfected with human L-FABP [16]. This upregulation was partially blocked using anti-TNF-α antibodies. Moreover, tubular L-FABP delayed the progression of glomerular damage by reducing oxidative stress and inflammatory mediators in a mouse model of IgAN [17]. However, only serum TNFR1 or TNFR2 were determinants of renal interstitial fibrosis in multiple regression analysis. Therefore, further studies are needed to investigate this further.

The primary focus of the current study was to examine the relationship between circulating or urinary excretion of TNFRs and the clinical or histological findings in IgAN patients. Nevertheless, our histological analyses revealed notable findings. DBP and eGFR were commonly correlated with interstitial fibrosis, tubular atrophy, and glomerulosclerosis.

There were several limitations to the current study. First, high CV of KIM-1 might be factored into the statistics, which showed no correlation between KIM-1 and eGFR, while all the other markers have significant negative correlations with eGFR. Second, urinary levels of TNFRs are increasing as eGFR decreases in IgAN patients, but this seems to be the opposite when taking normal controls into account. We estimate that within normal renal function range, considerable individual variability in the levels of urinary TNFRs is thought to exist compared with the levels of serum TNFRs. In addition, the small numbers of healthy subjects might have affected the result. Third, the study cohort was limited to relatively less advanced cases. Fourth, because most renal biopsy specimens did not include the renal medulla, the expression of TNFRs in the renal medulla could not be evaluated. Therefore, we could not rule out the possible association between serum TNFR levels and the protein levels of TNFRs in the renal medulla. Moreover, immunohistochemistry for TNFR was performed in only limited number of biopsy samples, and therefore, the results might not be reflective of all the study patients. Fifth, because transforming growth factor-β and connective tissue growth factor were not measured, we could not compare the usefulness of measuring serum TNFRs with existing markers. Finally, the causal association between serum TNFRs and renal interstitial fibrosis remains unclear because of the cross-sectional design, which limits the conclusions that can be drawn regarding the mechanism or temporal relationship. A prospective serial renal biopsy study would provide information regarding how these biomarkers might affect renal interstitial fibrosis over time; however, such a study is ethically questionable.

In conclusion, elevated levels of serum TNFRs at the time of renal biopsy are significantly associated with the severity of renal interstitial fibrosis in IgAN patients.

## Supporting Information

S1 Table Clinical characteristics, levels of inflammatory markers, and histological findings in selected IgAN patients who had levels of serum TNFR2 that ranked in the lowest 10 and highest 10. (XLSX)

S2 Table Spearman correlation coefficients between impaired renal function, renal TNFRs expressions, and their levels in serum and urine in IgAN patients. (XLSX)

S1 Fig Immunohistochemical staining for TNFR1 and TNFR2 with and without nuclear staining in the kidneys. Images were captured at 200× magnification. Images show nuclear staining without TNFR immunostaining in the glomeruli (C, F). TNFR1 and TNFR2 immunostaining without nuclear staining show unstained regions that are expected to be the nucleus [TNFR1, B; TNFR2, E (arrows)]. The TNFR-stained regions are also expected to be the nucleus [TNFR1, A; TNFR2, D (arrows)] and might represent staining in the supranucleus cytoplasm.
(EPS)
